# A snapshot of the organization and provision of primary care in Turkey

**DOI:** 10.1186/1472-6963-11-90

**Published:** 2011-05-04

**Authors:** Dionne S Kringos, Wienke GW Boerma, Ernst Spaan, Martina Pellny

**Affiliations:** 1 NIVEL-Netherlands Institute for Health Services Research, Otterstraat 114-118, 3500 BN Utrecht, The Netherlands; 2 Radboud University Nijmegen Medical Center, PO Box 9101, 6500 HB Nijmegen, The Netherlands; 3 World Health Organization, Level 4, Provident Plaza One, Downtown Boulevard, 33 Ellerly Street, Suva, Fiji

## Abstract

**Background:**

This WHO study aimed to support Turkey in its efforts to strengthen the primary care (PC) system by implementing the WHO Primary Care Evaluation Tool (PCET). This article provides an overview of the organization and provision of primary care in Turkey.

**Methods:**

The WHO Primary Care Evaluation Tool was implemented in two provinces (Bolu and Eskişehir) in Turkey in 2007/08. The Tool consists of three parts: a national questionnaire concerning the organisation and financing of primary care; a questionnaire for family doctors; and a questionnaire for patients who visit a family health centre.

**Results:**

Primary care has just recently become an official health policy priority with the introduction of a family medicine scheme. Although the supply of family doctors (FDs) has improved, they are geographically uneven distributed, and nationwide shortages of primary care staff remain. Coordination of care could be improved and quality control mechanisms were lacking. However, patients were very satisfied with the treatment by FDs.

**Conclusions:**

The study provides an overview of the current state of PC in Turkey for two provinces with newly introduced family medicine, by using a structured approach to evaluate the essential functions of PC, including governance, financing, resource generation, as well as the characteristics of a "good" service delivery system (as being accessible, comprehensive, coordinated and continuous).

## Background

The background to primary care reforms differs between Western Europe and the countries of Central and Eastern Europe and those formerly belonging to the Soviet Union. In the first mentioned countries emphasis on primary care (PC) is a response to rising costs and changing epidemiological and demographic trends, while the second mentioned countries are struggling to improve the performance of their health care systems as a whole [1-7].

The WHO World Health Report "Primary health care - now more than ever" has therefore stressed the need to bring responsive health services closer to the population, to provide people-centred care and to produce knowledge on how to best organize PC [8]. So far, health care reforms are often insufficiently based on evidence. Nowadays, however, policy makers and managers increasingly demand evidence of progress in reforms and strategies to make services more responsive to changing patient needs and demands [3,6-8]. This implies also that professionals who work in PC as well as their patients need to be heard by letting them evaluate access, coordination and convenience of services [9].

### WHO Primary Health Care Programme

The World Health Organization (WHO) Regional Office for Europe supports 53 member states to strengthen their health care systems. Since 2000, WHO has increased its focus on how health systems are governed, how they are provided with human and other resources, how the funding of health influences access to services and how the provision of services is organized and implemented. The latter is especially important in PC since in many member states knowledge on this is still scarce. WHO Europe therefore offers support to increase this evidence by means of Biennial Collaborative Agreements (BCAs), which are joint agreements between the respective Ministry of Health and WHO. Turkey started in the mid-90s with a major health care reform restructuring the primary care services offered [10,11]. As a result in 2007, the Ministry of Health in Turkey expressed the wish to support their primary care reforms by implementing the WHO Primary Care Evaluation Tool. Subsequently, WHO Europe commissioned its Collaborating Centre NIVEL to implement the Tool in order to gather survey-based data on the progress of the reform - complementing existing routine data - making it possible to better inform further policy decisions aimed at strengthening PC.

This article provides an overview of the findings on the organization and provision of PC in Turkey in 2007-2008.

## Methods

### Study design

**Figure 1:**
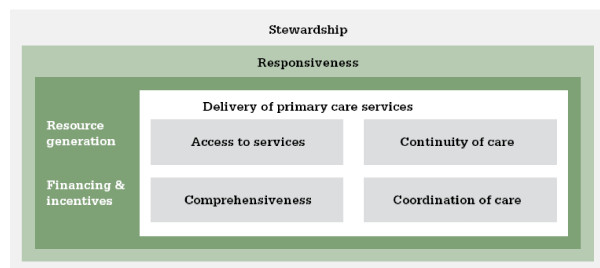
**Primary care evaluation framework**.

The WHO Primary Care Evaluation Tool (PCET) was implemented in two provinces (Bolu and Eskişehir) in Turkey in 2007/08. Figure 1 shows the Primary Care Evaluation Framework on which the PCET is based.

The PCET consisted of three instruments to evaluate the complexity of the PC system: a checklist of questions concerning the health system and status of PC at national level; a questionnaire for family doctors (FDs) and a questionnaire for their patients. The questionnaires were tailored to the Turkish situation of PC based on preparatory communications with policy makers and FDs, and a country visit of the research team to the provinces Bolu and Eskişehir.

The national level questionnaire contained 54 (prestructured and open ended) questions covering: PC policy, legislation and rules, workforce volume, training and education, health financing and coordination. An annex was also included, with 14 questions on statistical data to be filled in by experts from the Ministry of Health covering: workforce supply, funding sources, health budgets and payments.

The questionnaires for FDs and patients were prestructured with precoded answers. The questionnaire for FDs included 54 questions on: basic provider information, including education, and professional association membership; location and number of patients covered by the practice; workload and practice staff; accessibility; quality improvement and research; patient information; employment and income of the provider; coordination and teamwork; and equipment and clinical tasks. The patient questionnaire included 25 questions covering: basic patient information; visiting frequency and continuity of care; payment for services; and the patient's opinion on access, responsiveness and quality of PC services as well as on the cooperation between health care providers.

Subsequently, the questionnaires were translated into Turkish in a check and double-check procedure.

During a second country mission of the WHO team, the implementation of the PCET was prepared with the local coordinator (Ministry of Health and their provincial administrations). Fieldworkers were trained, for instance with regard to the confidential nature of the questionnaires. Agreement was reached on the details of the sampling and recruitment procedure, and details of the data collection strategy and logistics were discussed. Analysis and reporting were carried out by the research team in the Netherlands.

### Bolu and Eskişehir

The Ministry of Health selected two areas for implementation of the PCET: the provinces of Bolu and Eskişehir. These two areas were regarded as being representative of Turkey as a whole. Turkey is in the middle of a transition towards a nationwide system of family medicine [10,11], and the two selected provinces had both implemented the new PC model, one province being more rural, one more urban. By early 2008, 13 Turkish provinces (out of 81), including Bolu and Eskişehir, had introduced the concept of family medicine. Training in family medicine was provided at 20 universities and involved a three-year specialization after graduation from the regular four-year medical training. Parallel to this, a retraining programme for physicians in general practice was developed with three phases: a ten-day retraining course; a one-year distance learning course and then three-years of training/practice on site. By May 2007, all general practitioners (GPs) and the auxiliary/practice nurses in 12 pilot provinces had completed the first phase. From early 2008, additional provinces began the transition to the new family medicine system.

Bolu province is situated in north-western Turkey in the Black Sea region and lies halfway between the large cities of Istanbul and Ankara. It covers an area of 7410 km^2 ^and has a population of 263 619 [12]. Bolu province is predominantly rural. Bolu town is the administrative centre of the province and, in 2004, had a population of 85 000. Eskişehir province, also in north-western Turkey, is more urbanized and industrialized. The provincial capital of Eskişehir had a population of around 700 000 in 2004.

### Sampling and data collection strategy

A random sample of FDs was taken, based on lists of active practitioners in Bolu and Eskişehir. Taking into account the available resources and capacities of the provincial health authorities, the following sample was decided upon: in Bolu, the total number of newly trained FDs was limited (69); one third of them (every third FD) was randomly included in the survey. The number of newly trained FDs in Eskişehir was larger (208), so a smaller proportion (one sixth) was included. This resulted in a total sample of 55 FDs for both provinces. It was agreed that, in case of illness or unavailability, the next FD on the list would be included. Trained fieldworkers visited these FDs to hand over the questionnaire.

For the patient survey, with each included FD a target of 20 completed questionnaires was set. To achieve this number the fieldworkers asked every patient that visited the practice to cooperate until the number of 20 was achieved. This resulted in a total of 1100 targeted patients (460 in Bolu; 640 in Eskişehir). For both FDs and patients, the actual response rate exceeded the minimum required sample size, because of the preferences of the local partner and the actual interest of health workers and patients in being included.

To safeguard confidentiality, the filled-in questionnaires were collected by the fieldworkers, checked for completion and then dispatched in a sealed envelope to the collection point at the national health administration. Thus, although the local health authorities had a facilitating role, they did not have direct access to the information provided by individual doctors and patients.

For the national level questionnaire, experts were identified by the local coordinator, representing the Ministry of Health, FD associations and medical chambers, health insurers, academics and consumer or patient organisations. After the experts had filled in the questionnaire individually, a joint meeting was organized to discuss and clarify answers and to reach consensus.

SPSS Data Entry Station version 3.0.3 was used for data entry. SPSS version 14 was applied for data analysis. Both were performed by the research team in the Netherlands.

A comprehensive description of the development of the PCET, and the applied methods are published elsewhere [13].

## Results

### Respondents

The national level questionnaire was completed by a panel of 11 experts from the government, universities and professional association. Statistical data have been provided by the Ministry of Health.

The pilot implementation included 78 FDs; 37 in Bolu and 41 in Eskişehir. In both provinces most physicians were from urban family health centres, but in Eskişehir this proportion was greater (81%) than in Bolu (68%). In both provinces two thirds of the FDs were male and one third was female. Respondents were relatively young; on average 36 years in Bolu and 41 in Eskişehir. Since family medicine had recently been introduced, physicians had little experience as FDs (on average 1.5 and 2.5 years in Bolu and Eskişehir respectively).

In total 1548 patients filled in a questionnaire; 738 in Bolu and 810 in Eskişehir. The average age was around 40 years and the majority were women. Almost half of the patients only had primary education. Almost all respondents lived in a family setting; living alone was extremely rare. Three quarters of patients were from urban family health centres.

Table 1 provides a summary overview of a selection of proxy indicators by PC function for Bolu and Eskişehir. We will discuss the main results in the next sections. Differences between the two provinces will be indicated where appropriate.

**Table 1:** Overview of selected proxy indicators by primary care function for Bolu and Eskişehir

Primary care functions	Selected proxy indicators	Findings FDs (N = 78) Patients (N = 1548)
Stewardship	Department in Ministry of Health (MoH) specifically dealing with primary care (PC)	yes
	% family health centres with patient complaint procedure in place	78%
Financing	Employment status of FDs	100% state employed
	Remuneration method	Capitation + Performance related payments
Resource generation	% of all active physicians working in PC *	13.8%
	% provinces with Family Medicine (FM) being introduced *	16%
	% FDs among all PC doctors in provinces with FM being introduced*	72%
	Average age of FDs	39 years
	Hours FDs spend on professional reading (per month)	9.5 hours
	% medical universities with department of family medicine*	74%
	Average number of items of medical equipment available to FDs (from a list of 29 items)	21 items
	% of FDs reporting no or insufficient access to a laboratory	3.8%
	% of FDs reporting no or insufficient access to X-ray equipment	45%
	% of FDs with a computer in the family health centre	97%
Access to services	% patients reporting copayments for drugs prescribed in PC	57%
	% of patients living within 20 minutes travel from PC facility	79%
	Average number of registered patients per FD	3715
	Average number of patient consultations per day	47
	Average number of home visits per day	1.7
	Average working hours of FD per week	46**
	Average length of patient consultations (minutes)	11
	Reported average utilization rate (frequency) by patient per year	7.6
	% of FDs using an appointment system	1%
Coordination of care	% of FDs sharing premises with other FD(s)	90%
	% of FDs having regular meetings with practice nurses	77%
Continuity of care	% FDs keeping medical records routinely	43%
	% of patients assigned to their FD (not chosen)	71%
	% of patients with their FD for at least 1 year	59%
Comprehensiveness	% of FDs frequently using clinical guidelines	16%
	Score for FDs' role in first contact care for a selection of 17 health problems (range of score 1 (never) - 4 (always))	2.47
	Score for FDs' involvement in the treatment of a selection of 18 diseases (range of score 1 (never) - 4 (always))	2.59
	Score for FDs' or team members involvement in the provision of a selection of 16 preventive and medical-technical procedures (range of score 1 (never) - 4 (always))	2.41
	% of FDs having regular meetings with local authorities	26%

*at national level

** the official minimum working hours of FDs are 40 hours. Additional hours worked is on a voluntary basis.

### Policy development (national level questionnaire)

PC was acknowledged as being important a long time ago in Turkey but its implementation has only recently become effective. The concept of integrated PC was introduced in 1961. Plans launched in the early 1990s, including those for decentralization, partial gatekeeping by FDs and better training programmes were not successful. It was not until 1996 that family medicine was adopted as a more comprehensive model for PC. Since 2003, this model has been implemented in 13 provinces (by early 2008) out of 81 overall in Turkey [14].

Despite decentralization, the role of the Ministry of Health in PC was still strong. To a large extent, the management and provision of PC services in Turkish provinces was uniform. The Ministry retains a strong influence on staff appointments at provincial health directorates and directorates take technical decisions in line with central guidelines. Furthermore, they assume major responsibilities for the management of estate and human resources in their province.

The government's vision of PC has been published in various laws and documents. These cover the specification of PC disciplines and their tasks and responsibilities, education and accreditation requirements, norms for availability of doctors and facilities, medical record requirements and requirements related to performance monitoring. Organizations of professionals and patients were rarely involved in the policy making process but rather in the implementation of policies [15]. It is expected that the roles (in the policy making process) of these organizations will become more formalized in future. The position and rights of patients was formally acknowledged but this position has not yet been fully translated into practice. For instance, patient complaint procedures were only present in 78% of the family health centres included in the family doctor survey.

Topics that were debated by health policy experts during the period of the survey's implementation were, for example, shortages of physicians and nurses and the (unequal) provincial distribution of physicians in the country; the improvement of coordination of care between levels of care through gatekeeping and multidisciplinary teams; and physical improvement of health care premises and equipment.

### Financial incentives for providers (national level and family doctor questionnaire)

Almost all FDs (90%) were state employed, receiving fixed salaries, with additional capitation elements of payment. This included bonuses for working in disadvantaged areas and additional payments for the performance of predefined preventive services. The recent introduction of a more performance-related payment scheme (mixed scheme) seemed to be a major step towards implementing a more comprehensive, efficient and responsive PC system. Incentives needed to be fine-tuned in order to avoid overproduction and to stimulate quality of care.

### Professional development (national level and family doctor questionnaire)

The implementation of family medicine in the 13 provinces (as of early 2008) was well underway. A total of 27 500 physicians were working in PC nationally. In these provinces with newly introduced family doctors, a majority of PC doctors were now FDs. Nationwide (81 provinces), however, the proportion of FDs was only 10%. Three quarters of all medical universities in Turkey had departments for family medicine where FDs were trained. However, the capacity in the residential programmes (about 500 places per year in 40 medical universities) was not fully used. Only 80% of places were filled. In light of the current severe shortages of physicians and nurses in PC, everything should be done to ensure full use of capacity. Registers of PC professionals were in place, but it was not clear whether they were up-to-date and how they were used for workforce planning.

There was a national organization of FDs (TAHUD) with a broad range of activities; however, its role in the policy-making process was not formalized. In addition, GP and FD organizations were developing in eight provinces in early 2008.

Many FDs (54% and 70% in Bolu and Eskişehir respectively) reported having difficulty in keeping up with the latest professional developments. FDs in Eskişehir reported spending much more time on professional reading (12 hours/month) than their colleagues in Bolu (7 hours/month).

### Quality management (national level questionnaire)

Quality improvement mechanisms such as obligatory re-certification schemes or periodic knowledge and skills tests were not yet in use and formalized. There were few requirements concerning the quality and confidentiality of medical records. Formal and informal mechanisms of performance assessment, such as practice inspections and medical audits, were infrequently applied. A positive change here may be the introduction of the performance-related payment scheme. However, its focus seemed to be more on the quantitative side of performance than on the quality of care. So far, clinical guidelines in PC were developed and implemented under the exclusive responsibility of the Ministry and drawn up by assigned medical specialists with minor inputs from FDs. The use of the guidelines was not formally evaluated. The responding FDs reported that they did not frequently use clinical guidelines.

### Financial and geographical access for patients (patient questionnaire)

Although PC was officially free of charge, this was not true for prescribed medicines or injections. Half of the patients reported co-payments for these services. Some patients (12%) also reported co-payments for home visits and for visits to a specialist after referral from the FD. Co-payments seemed to be an obstacle to the utilization of health care services. A significant minority of patients answered they had abstained from a visit to their FD (9%) or a medical specialist (17%) for financial reasons.

The national level questionnaire showed that PC physicians were unevenly distributed between provinces in Turkey. This suggested provincial differences in the availability of PC services. In Bolu and Eskişehir, however, patients had no difficulty to reach family health centres, pharmacies and hospitals.

### Organizational access to services (family doctor and patient questionnaires)

Compared to the European situation, practices were very large with an average of 2484 people per physician in those provinces where family medicine implementation had not yet started, but there were also variations across the country. In provinces where the family medicine reforms had already been implemented, the average population per family physician was around 3500; in Bolu and Eskişehir for example the average was 3700. As a result, the number of consultations per day was high (47 on average). Home visits were rarely made (on average 1.7 per day).

Most patients were satisfied with the current opening hours of the family health centres (84% on average; which varied significantly between centres), the availability of medical staff during these hours (83%) or getting the opportunity to speak to a doctor on the telephone (49%). Almost all patients reported that it was usually possible to visit a FD the same day and waiting times were acceptable, even if making an appointment was unusual. Visiting a FD outside the normal office hours, in the evening or on weekend, was only rarely possible.

Family health centres hardly ever used the Internet for their communication with and information to the patients. Consultation time per patient was relatively short (11 minutes on average).

### Cohesion within primary care (family doctor questionnaire)

Lack of coordination of care seemed to be a major problem. For instance, multidisciplinary teamwork, for the benefit of patients with chronic diseases (such as diabetes) hardly existed in Bolu and Eskişehir. The majority of FDs worked in teams of three or more FDs. In addition to FDs, family health centres consisted of practice nurses and, in most cases, midwives as well. Other PC disciplines, like physiotherapists, dentists and pharmacists were not usually part of the family health centre. Cooperation was not strongly formalized between team members. Regular meetings were not usual between FDs and nurses and even less so between FDs and midwives.

### Coordination with other care levels (family doctor and patient questionnaires)

There were no mechanisms to promote coordination between the primary and the secondary care levels. The policy on the gatekeeping role for FDs was not clear to patients and, in daily practice, gatekeeping was not well maintained. Despite this lack of clarity, most people first visited their FD with new health problems. It was not usual to refer patients back to PC after hospitalization. Working relations between FDs and medical specialists and hospitals left much to be desired. Consultation or asking advice from medical specialists were infrequently reported (on average only by 8% and 4% of the FDs in Bolu and Eskişehir respectively).

Referral letters were poorly used (only frequently used by 56% and 12% of the FDs in Bolu and Eskişehir respectively) and medical specialists did not inform FDs properly about their treatment. Discharge reporting was not formalized.

### Informational continuity (family doctor and patient questionnaires)

Conditions for clinical and other information were good in the family health centres. 97% of respondent FDs had a computer at their disposal, which was used for keeping patients' medical records.

However, these possibilities were not optimally used because records were not kept routinely. Furthermore, it was difficult to use computer records to produce lists of patients on the basis of common diagnosis or elevated health risks. Most patients (45% on average) were not sure whether they could see their own medical files if they wanted. Many patients (63% on average) felt the exchange of information between physicians could be better. Patients' expectations of the communication between their own FD and other physicians were also modest.

### Longitudinal continuity (patient questionnaire)

Patients had visited the family health centres about seven times during the previous year according to their estimates. In Eskişehir, the visiting rate with FDs was much higher in the urban family health centres than in the rural ones. Patients thought that it was not possible to choose their doctor. They had usually been assigned to their current FD.

Patients saw restrictions in changing from one doctor to another. Since family medicine had been introduced rather recently, patients had been with their doctors for a rather short period.

### Interpersonal continuity (family doctor and patient questionnaires)

Despite the fact that FDs worked in groups, patients would generally (93%) see their own FD during each visit. Consultations were relatively short and FDs did not always have the patient's medical file at hand.

Patients were satisfied about the way they were treated by their FD (95%), although they were not generally convinced that the FD was aware of their personal situation (40%) and the details of their medical history (44%). Patients found that FDs took sufficient time (86%), listened (94%) and communicated (90%) well and kept to promises and appointments (84%). Patients were reserved about their FD's preparedness to make a home visit (45% of patients did not know). Many patients (40%) were also not sure if their FD would be the right person for discussing non-medical problems that impacted on health.

### Services delivery (family doctor and patient questionnaires)

FDs had a strong position as the doctor of first contact for health problems of children (except for hearing problems), and women (except for menstruation problems). For problems with strong social and psychological components, the first contact role was less developed.

For sexual problems, psychiatric or relationship problems, FDs were not the first choice to contact. FDs reported to be moderately involved in the provision of preventive care and medical technical procedures. Expansion of these tasks could include insertion of intrauterine devices and minor surgical procedures.

Activities of FDs aimed at specific patient groups or other public health related tasks mainly covered the areas of mother and child health and family planning. FDs did not conduct much screening for sexually transmitted infections, HIV/AIDS, tuberculosis or cervical cancer.

Family health centres were reasonably well equipped, especially with computers. With regard to medical equipment, the situation in Eskişehir was slightly better than in Bolu. Typically absent were peakflow meters, tuning forks and ultrasound equipment. FDs in Eskişehir were better equipped for gynaecological services than their colleagues in Bolu. A general problem as perceived by FDs was insufficient access to external X-ray facilities; access to laboratory facilities however was felt to be very good.

Links with the community turned out to be weak. FDs in Bolu mentioned community connections more frequently than FDs in Eskişehir, e.g. meeting with local authorities, social workers or religious groups.

## Discussion

Based on the results of this study, a number of recommendations can be made to further improve and strengthen PC in Bolu and Eskişehir specifically, and at national policy level in Turkey.

At national level, organizations of professionals and patients were already involved in the policy making process but rather on an ad-hoc basis. The inclusion of stakeholders on a more formal basis for example in a standing committee or by officially delegating health policy and implementation responsibilities to them can strengthen the effectiveness and responsiveness of primary care policies.

The important role and position of patients was formally acknowledged at national level, but the questionnaires showed that patients were not always aware of their rights and the functioning of the new system, nor did patients and FDs realize fully the potential of informed and active patients for better health outcomes. A public information campaign targeting the population as well as physicians with differentiated messages on the rules of the new system could be beneficial, at least in the two included provinces.

Much has been done since the start of the reforms - however, nationwide the proportion of family doctors to other specialities was still only 10%. It can be considered to fully use existing capacities in the residential programmes (about 500 places per year in 40 medical universities; but only 80% were used) - and to expand this capacity in the whole country.

It is recommended to continue with the new payment scheme that keeps family medicine attractive for new students and to consider adding other incentives such as free internet connections and e-learning programs for doctors in rural areas. The reputation of FDs can be enhanced by subsidizing and supporting research for FDs (for example in drawing up clinical guidelines) or extending the task profile of FDs.

Even though the involvement of FDs in the treatment of diseases could be improved if compared to that of colleagues in Europe, in comparison with the results of a European study conducted 15 years ago [16], the current situation in Turkey has very much improved. However, attention needs to be paid to the size of practices and daily workload of FDs, so that they have more time for inter-collegial consultations, home visits, recordkeeping and skill upgrading. With an average of 3700 patients, family practices were very large, in comparison to practices of family doctors in all other European countries (e.g. the average patient list of a general practitioner in the Netherlands counts 2322 patients, in Poland 1539 and in Italy 1094 patients).

It is advisable to keep the register of PC professionals up-to date and to use it for active human resources planning.

Formalized multidisciplinary team work within PC or between levels of care for the benefit of for example patients with chronic diseases or multi-morbidities hardly existed in Bolu and Eskişehir. Referral letters were poorly used by the responding FDs and secondary specialists were not informing FDs routinely about their treatment. Discharge reporting from the hospital was not formalized in either one of the two provinces. It may be considered to introduce clear reporting rules and link it to the new IT software and by doing so, enhance the coordinating role of the FD. Furthermore, team working schemes for the core PC team and providing training on it could also be considered.

The introduction of new disciplines in PC such as nurse practitioners and others that can support the network of an extended family practice model, or include existing ones more closely, for example pharmacists, physiotherapists and dentists may be considered. Coordination between health and social services can be enhanced by stimulating stronger links between primary health care facilities and the community.

The introduction of performance elements into the payment scheme for FDs has been a successful first step, however with too much impact on quantity and little on quality. A national strategy may be considered to systematically establish quality improvement mechanisms: certification and re-certification schemes, continuous medical education programs based on the need of doctors, practice inspections and medical audits, peer review circles, routine electronic patient records, and the participation in the development of clinical guidelines. Obviously priorities need to made in implementing these recommendations. To achieve sustainable improvements at primary care process and outcome level, it is advised to start with recommendations concerning the structure level of the health care system that set the conditions for the delivery of care process (e.g. training and education, human resource planning, financial incentives), before implementing practice level recommendations (directly relating to the delivery of care process).

### Strength and Limitations

A major strength of this study is that it provides a systematic baseline assessment of the current state of PC in two provinces of Turkey that can be further used for intra/inter-country comparisons and identification of good practices. In addition, the impact of this study goes beyond collecting data, also shown by the exceeded planned response rate. The introduction of the activities at central, provincial and local level implied information transfer (e.g. sharing of experiences) and raising awareness on issues of organization and provision in PC and the identification of ways to improve PC. For example, the questionnaires were adapted to the country situation together with a national working group, providing room to discuss unclear questions and concepts and to give ownership of the PCET to the group. The application of the survey-based tool was also likely to increase the motivation for self-assessment of policymakers and providers.

An important limitation of the study is that the instrument relies on self reports, rather than on direct observations or registrations. Despite measures introduced in the PCET, there may still be answering bias. For practical reasons, such as time and funding, the study was limited to two out of 81 provinces in Turkey. Another limitation, is the non random sampling of patients. The first 20 patients that visited a certain family health centre on a normal working day were included, which may have caused an overrepresentation of patients that usually visit FDs in the morning (e.g. mothers with young children, or retired persons).

## Conclusions

The study provides a valuable overview of the current state of PC in two provinces in Turkey with newly introduced family medicine by using a structured approach to evaluate the essential functions of PC, as indicated in the WHO health systems framework, including governance, financing, resource generation, as well as the characteristics of a "good" service delivery system (as being accessible, comprehensive, coordinated and continuous).

## Competing interests

The authors declare that they have no competing interests.

## Authors' contributions

DK, WB and ES performed the study in collaboration with MP. DK wrote the manuscript. WB, ES and MP reviewed drafts of the manuscript. All authors read and approved the final manuscript.

## Pre-publication history

The pre-publication history for this paper can be accessed here:

http://www.biomedcentral.com/1472-6963/11/90/prepub
